# The molecular dialogue between *Arabidopsis thaliana* and the necrotrophic fungus *Botrytis cinerea* leads to major changes in host carbon metabolism

**DOI:** 10.1038/s41598-017-17413-y

**Published:** 2017-12-07

**Authors:** Florian Veillet, Cécile Gaillard, Pauline Lemonnier, Pierre Coutos-Thévenot, Sylvain La Camera

**Affiliations:** 1 Laboratoire Ecologie et Biologie des Interactions, Equipe “SEVE-Sucres et Echanges Végétaux-Environnement”, Université de Poitiers, UMR CNRS 7267, F-86073 Poitiers, France; 2 Present Address: Department of Plant Biology and Institute for Genomic Biology, University of Illinois at Urbana-Champaign, Urbana, Illinois 61801 USA

## Abstract

Photoassimilates play crucial roles during plant-pathogen interactions, as colonizing pathogens rely on the supply of sugars from hosts. The competition for sugar acquisition at the plant-pathogen interface involves different strategies from both partners which are critical for the outcome of the interaction. Here, we dissect individual mechanisms of sugar uptake during the interaction of *Arabidopsis thaliana* with the necrotrophic fungus *Botrytis cinerea* using millicell culture insert, that enables molecular communication without physical contact. We demonstrate that *B. cinerea* is able to actively absorb glucose and fructose with equal capacities. Challenged *Arabidopsis* cells compete for extracellular monosaccharides through transcriptional reprogramming of host sugar transporter genes and activation of a complex sugar uptake system which displays differential specificity and affinity for hexoses. We provide evidence that the molecular dialogue between *Arabidopsis* cells and *B. cinerea* triggers major changes in host metabolism, including apoplastic sucrose degradation and consumption of carbohydrates and oxygen, suggesting an enhanced activity of the glycolysis and the cellular respiration. We conclude that beside a role in sugar deprivation of the pathogen by competing for sugar availability in the apoplast, the enhanced uptake of hexoses also contributes to sustain the increased activity of respiratory metabolism to fuel plant defences.

## Introduction

The coevolutionary arm race between plants and pathogens led to the development of complex molecular mechanisms for perception and defence activation against the invader^1^. Plants initiate basal defence against pathogens upon the recognition of conserved Pathogen-Associated Molecular Patterns (PAMPs) by Pattern-Recognition Receptors (PRRs)^2^. This PAMP-Triggered Immunity (PTI) helps to limit the spread of the disease^3^. In some cases, pathogen effectors are recognized by specific intracellular disease resistance proteins promoting an immune response called Effector Triggered Immunity (ETI)^4^. Although immune responses are faster, more robust and prolonged in ETI than in PTI, they share common features for danger perception and defence activation^5,6^. Pathogens can be classified according to their infection and feeding strategy. Biotrophic pathogens feed on living tissues forming specialised structures known as haustoria, necrotrophs kill host cells and acquire nutrients from dead tissues, while hemibiotrophs have an intermediate lifestyle^7–9^. The pathogenicity of *Botrytis cinerea*, a model for necrotrophic fungi^10^, involves a large array of molecules, *e.g*. ROS, toxins, oxalic acid, but also numbers of secreted proteins including proteases, plant cuticle and cell wall degrading enzymes or necrosis-inducing factors^11^. Some of them are required for full virulence and/or actively release host-derived signals, indicated as Damage-Associated Molecular Patterns (DAMPs)^12^. The PRR-mediated detection of fungal PAMPs, (*e.g*. chitin) and DAMPs (*e.g*. oligogalacturonides), is accompanied by the activity of additional receptor-like kinases to transduce the signal and to induced host defence responses, such as ROS production, callose deposition, MAP kinase activation, hormone and phytoalexin production, defence gene expression and HR-like cell death^13–15^.

Pathogens use contrasting mechanisms of nutrient acquisition to ensure a continuous supply of carbohydrate from their host^16–20^. A direct competition occurs at the plant-pathogen interface where activities of sugar transporters from both pathogens and plants seem to be critical for the outcome of the interaction. The virulence of the corn smut fungus *Ustilago maydis* is associated with the activity of the plasma membrane-localised sucrose specific transporter (UmSrt1)^21^, which exploits the apoplastic sucrose resource from maize SUT1^22^. Bacterial pathogens manipulate the host sugar efflux machinery and take advantage of the nutrient niche created by the leakage of host sugars into the apoplast. For instance, *Xanthomonas* bacteria secrete TAL (transcription activator-like) effector proteins to induce the expression of sugar efflux transporters belonging to the SWEET family (Sugars Will Eventually be Exported Transporters)^23–25^. In return, plants can retrieve sugars from the infection niche through the activation of high-affinity sugar transporters. The induction of members of the Sugar Transport Protein (STP) family has been reported in response to fungal and bacterial pathogens, *e.g*. AtSTP13 in *Arabidopsis* and STP13 homologues in wheat (Lr67) and grapevine (VvHT5)^26–29^. AtSTP13 contributes to the basal resistance against *B. cinerea* and is required for antibacterial defence^27,29^. Recently, Yamada, *et al*.^29^ showed that AtSTP13 is associated with the PTI machinery since AtSTP13 interacts with PRR complexes. Accordingly, the STP13-mediated absorption of apoplastic hexoses seems to participate in the starvation of extracellular pathogens by restricting carbohydrate availability. Conversely, wheat STP13 homologue defective in sugar transport activity confers an enhanced resistance to wheat pathogens^28^. Thus, the STP13-mediated hexose uptake may rather be beneficial for biotrophs since intracellular hexoses are an essential reservoir of nutrients for haustorium-forming pathogens^16^.

Pathogen infections generate important modification of the host primary metabolism^30,31^. The reduction of photosynthesis and the accumulation of hexoses in the apoplast are general responses to pathogens, which often leads to a source/sink transition of infected tissues. The catabolism of soluble sugars is essential to provide source of carbon and energy, for the production of secondary metabolites, the reinforcement of the cell wall and signalling^32^. The coordinated activity of sucrose cleavage by cell wall invertases and host hexose transporters has been reported in several plant-pathogen interactions^33^. For instance, *Arabidopsis AtSTP4*/*Atβfruct1* and grapevine *VvHT5*/*VvcwINV* pairs are induced in response to biotrophic fungal infection^26,34^. AtCWIN1 was also responsible for the *Botrytis*-induced apoplastic invertase activity in leaves^35^. Because sugars seem to be preferentially taken up in the form of hexoses, pathogens have also evolved mechanisms to cleave extracellular sucrose and to gain access to released hexoses creating a flux of sugars from the host toward the pathogen^35–40^. However, their contributions to the pathogenicity and virulence is poorly described.

Our aim was to study the competition for sugars which takes place at the interface between *A. thaliana* and the necrotrophic fungus *B. cinerea*. To gain insight on how plant and pathogen cells compete for apoplastic sugars, we developed the millicell system, which is an innovative system allowing the molecular interaction between living organisms without physical contact. Therefore, we were able to study *Arabidopsis* and *Botrytis* responses separately and provide evidence for glucose and fructose uptake capacities in both partners. We pointed out a complex low and high affinity sugar transport system in *Botrytis*-challenged cells highlighting the importance of the extracellular hexose retrieval for the outcome of the interaction. Our study does not only provide insight into the regulation of sugar transport activity but also contributes to better understand modifications occurring in host carbon metabolism during PTI. We conclude that beside a role in sugar deprivation of the pathogen by competing for sugar availability in the apoplast, the enhanced uptake of hexoses also contributes to sustain the increased activity of respiratory metabolism to fuel plant defence.

## Results

### Establishing the molecular dialogue between *Arabidopsis* cell suspension culture and *B. cinerea*

**Figure 1:**
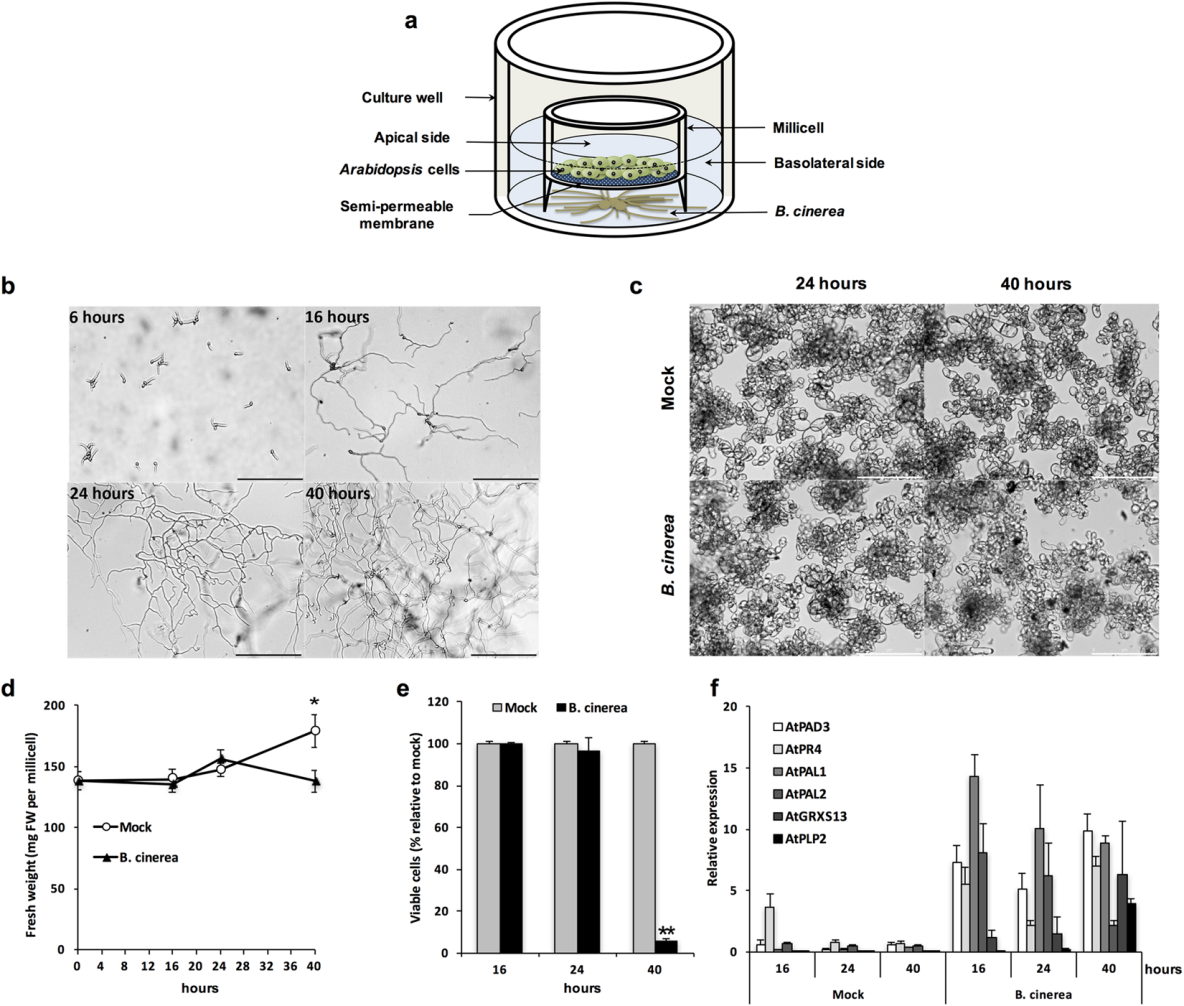
Establishment of the molecular dialogue between *Arabidopsis* and *B. cinerea* cells in the Millicell system. (**a**) Schematic representation of the Millicell system allowing the co-culture of *Arabidopsis* cells (apical side) and *B. cinerea* (basolateral side) through a hydrophilic PTFE cell culture insert. *Arabidopsis* cell suspension was grown up to the exponential phase of growth. After 4 days, cells were washed and resuspended in sucrose-containing medium. At time 0, a conidia suspension of *B. cinerea* was placed in a 6-well culture plate containing Millicell inserts with *Arabidopsis* cells in the apical compartment. In mock conditions, *Arabidopsis* cells were cultured without conidia in the basolateral side of the Millicell. (**b**)(**c**) Time course study of the morphological development of *B. cinerea* (**b**) and *Arabidopsis* cells (**c**) in the Millicell. Light microscopy observations were made after 6, 16, 24 and 40 hours for *B. cinerea* and after 24 and 40 hours for *Arabidopsis* cells. Scale bar = 250 μm. (**d**) Fresh weight of *Arabidopsis* cells at different times after culture initiation in the Millicell. Cells grown in Millicell were collected at indicated time points and fresh weight (FW) was measured. Data represent mean (+/−SE) of at least 4 for independent experiments. (**e**) Viability (MTS Tetrazolium-based assay) of *Arabidopsis* cells grown in the Millicell. Data are relative to the mock condition and represent mean (+/−SE) of at least 2 independent experiments. (**f**) Relative expression level of several defence-related and *Botrytis*-responsive genes in *Arabidopsis* cells. Gene expression was normalized to the plant reference gene *At4g26410*. Data represent mean (+/−SE) of at least 3 independent experiments. For (**d**) and (**e**), asterisks represent significant differences compared to the corresponding mock condition (Student’s *t*-test, * *P* < 0.05; ** *P* < 0.01).

In order to distinguish specific responses between host and *B. cinerea* cells, heterotrophic *Arabidopsis* cultured cells and fungal mycelium were cultivated on opposite sides of a Millicell culture plate insert, a hydrophilic PTFE permeable membrane with 0.4 μm pore size (Fig. 1a). The Millicell insert physically separates growing *Arabidopsis* cells and *Botrytis* conidia, which are trapped into basolateral and apical compartments, respectively (Fig. 1b,c). In the compartment containing *Botrytis* conidia, germ tubes were visible within 6 hours and mycelium fully covered the well after 40 hours (Fig. 1b). To ensure that the molecular dialogue was effective, we monitored several host cell responses during the course of the interactions. The growth of both mock and *Botrytis*-challenged cell suspensions started with an initial phase of latency (from 0 to 16 hours) followed by an increase of the biomass within 24 hours (Fig. 1d). At 40 hours, the fresh weight (FW) of the mock-treated cell suspension was 30% higher than the initial FW, whereas the growth of *Botrytis*-treated cells was stopped (Fig. 1d). This result indicates that the perception or the activity of molecules secreted by *B. cinerea* affected the proliferation of challenged cells. As we did not observe any obvious morphological differences between mock and *Botrytis*-treated cells after 24 and 40 hours (Fig. 1c), we evaluated the cell viability by a MTS Tetrazolium-based assay. After 16 and 24 hours, cell viability was affected neither in mock nor in *Botrytis-*treated cells (Fig. 1e). In contrast to the mock condition, only 5% of *Botrytis*-challenged cells were found to be metabolically active after 40 hours, suggesting that those cells were probably in the process of cell death.

To demonstrate that *Arabidopsis* cells perceived signals released by *B. cinerea* and triggered PAMP-induced responses, we monitored the expression of a set of defence-related genes. The expression of *AtPAD3*, which is required for the biosynthesis of the phytoalexin camalexin and for the appropriate defence against *B. cinerea* ^41^, is strongly induced upon *Botrytis* challenge (Fig. 1f). Consistently with previous studies, *AtPR4*, *AtPAL1* and *AtPAL2* were up-regulated with different expression patterns^42^ (Fig. 1f). The transcript accumulation of two *Botrytis*-responsive genes required to facilitate *B. cinerea* colonization, *i.e. AtPLP2* ^43^ and *AtGRXS13* ^44^, was clearly enhanced in challenged cells (Fig. 1f).

Taken together, these data showed that we were able to set up a molecular dialogue between *Arabidopsis* and *Botrytis* cells grown without any physical contact, using the Millicell insert. It likely involves the perception of PAMPs, DAMPs or effectors from *B. cinerea*, which is sufficient to elicit similar responses to those observed in infected leaves.

### Glucose and fructose are actively transported into *B. cinerea* mycelium

**Figure 2:**
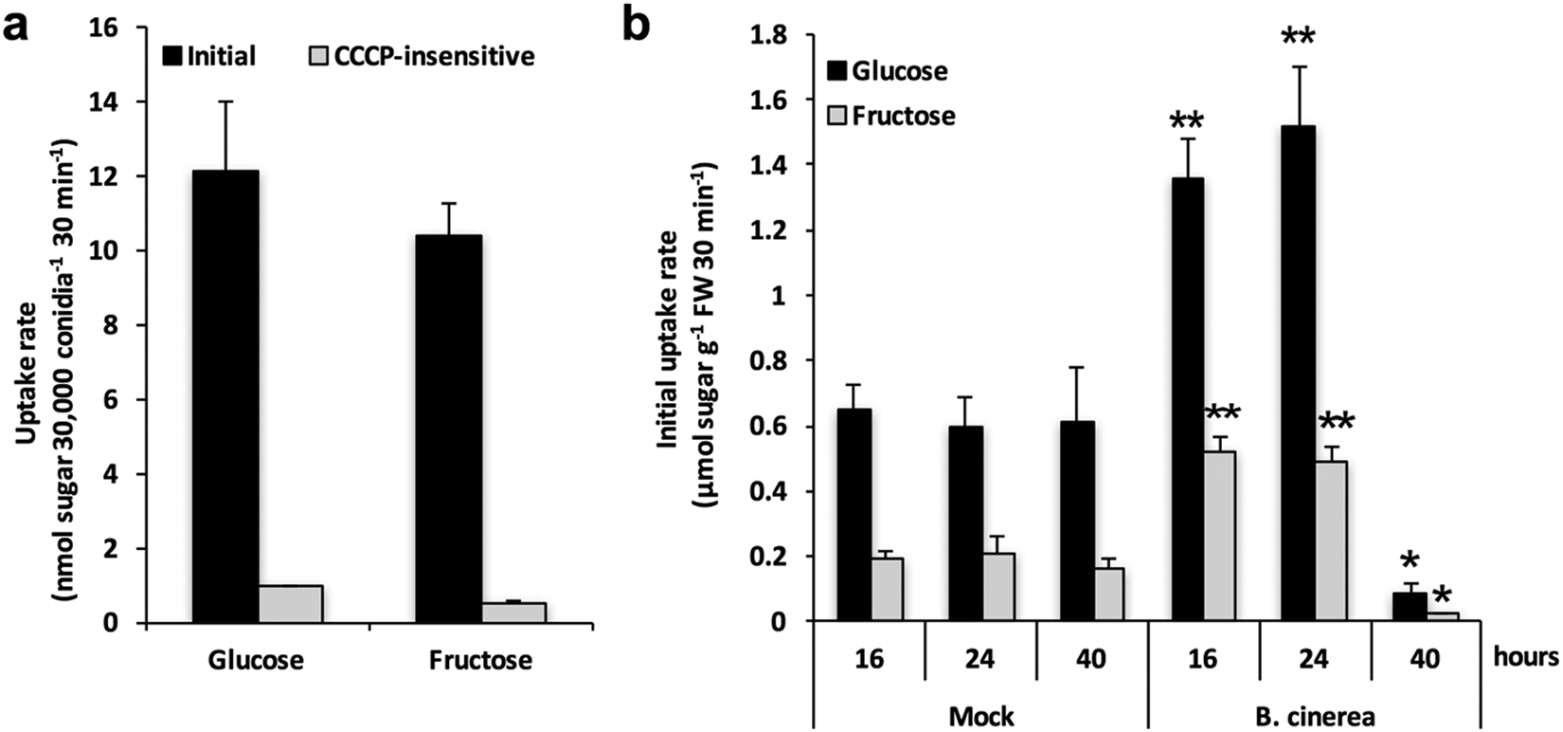
Hexose uptake into *B. cinerea* mycelium (**a**) and *Arabidopsis* cells (**b**) grown in the Millicell system. Thirty thousand conidia were cultured into the basolateral side of the Millicell in the presence of *Arabidopsis* cells into the apical side. Fungal mycelium and *Arabidopsis* cells were collected at indicated time points and incubated with 0.2 mM D-[^14^C]glucose or D-[^14^C]fructose. (**a**) Initial uptake rates of [^14^C]-labelled sugars by *B. cinerea* cells were determined 24 hours following the beginning of the interaction. To evaluate the CCCP-insensitive uptake rates, 20 μM CCCP was added to the reaction mixture 10 minutes before addition of labelled sugars. (**b**) Initial uptake rates of [^14^C]glucose and [^14^C]fructose by *Arabidopsis* cells were measured 16, 24 and 40 hours following the beginning of the elicitation with *B. cinerea*. Data represent mean (+/−SE) of at least 3 independent experiments. Asterisks represent significant differences compared to the corresponding mock condition (Student’s *t*-test, * *P* < 0.05; ** *P* < 0.01).

We took advantage of the molecular communication established between both partners to study their sugar absorption capacities. After 24 hours of co-culture of *B. cinerea* with *Arabidopsis* cells in the millicell, growing fungal mycelium was directly incubated with D-[^14^C]Glucose or D-[^14^C]Fructose. As shown in Fig. 2a, glucose and fructose were taken up by fungal mycelium with similar rates, 12 ± 1.9 nmol 30 min^−1^ and 10 ± 0.9 nmol 30 min^−1^, respectively. Glucose and fructose absorption into the mycelial suspension was almost totally inhibited in the presence of Carbonyl Cyanide m-Chlorophenyl Hydrazone (CCCP), a protonophore that destroys the transplasmalemma proton gradient (Fig. 2a). These results indicate that the growing mycelia of *B. cinerea* cultured in the presence of *Arabidopsis* cells is able to absorb either glucose or fructose mainly through a mechanism involving a proton gradient across the plasma membrane. In addition, we did not see any difference of the hexose uptake into *Botrytis* mycelia grown in the absence of *Arabidopsis* cells (data not shown), indicating that the capacity of *B. cinerea* to absorb hexoses is not affected by molecular signals potentially released by the host.

### *Arabidopsis* cells co-cultured with *B. cinerea* in the Millicell system display enhanced glucose and fructose uptake rates

*Arabidopsis* cell suspension treated with or without the growing mycelium of *B. cinerea* was further incubated with ^14^C-labelled glucose or fructose for uptake assays (Fig. 2b). Glucose uptake rates were approximately 3-fold higher than fructose uptake rates (Fig. 2b). CCCP inhibited about 80–90% of total uptake into elicited and non-elicited cells, pointing out an active transport system for those sugars (Supplementary Table 1). In non-elicited cells, initial uptake rates of both glucose and fructose were unchanged over time, indicating that experimental conditions did not affect cell homeostasis. Elicitation by *B. cinerea* had a significant impact on sugar transport since glucose and fructose uptake rates were induced approximately 2.5-fold after 16 and 24 hours (Fig. 2b). By contrast, a dramatic collapse of the sugar uptake rate was observed 40 hours after elicitation (Fig. 2b and Supplementary Table 1), which is consistent with MTS assays showing that only 5% of cells were viable (Fig. 1e). At this particular stage of the interaction, we demonstrated that elicited cells were transcriptionally active (Fig. 1f), but we assume that cells must have sustained damages, such as membrane permeabilization, that would impair sugar transport. To validate our assumption, we performed an absolute quantitation of DNA content in cells. We determined that DNA content is not affected by our experimental conditions (Supplementary Fig. 1), particularly in cells elicited for 40 hours, indicating that they did not reach the stage of the nuclear disassembly. Cells elicited for 48 h are not able to incorporate the vital stain neutral red in vacuoles (Supplementary Fig. 2), suggesting that the cell death occurs between 40 and 48-hours post-elicitation.

**Figure 3:**
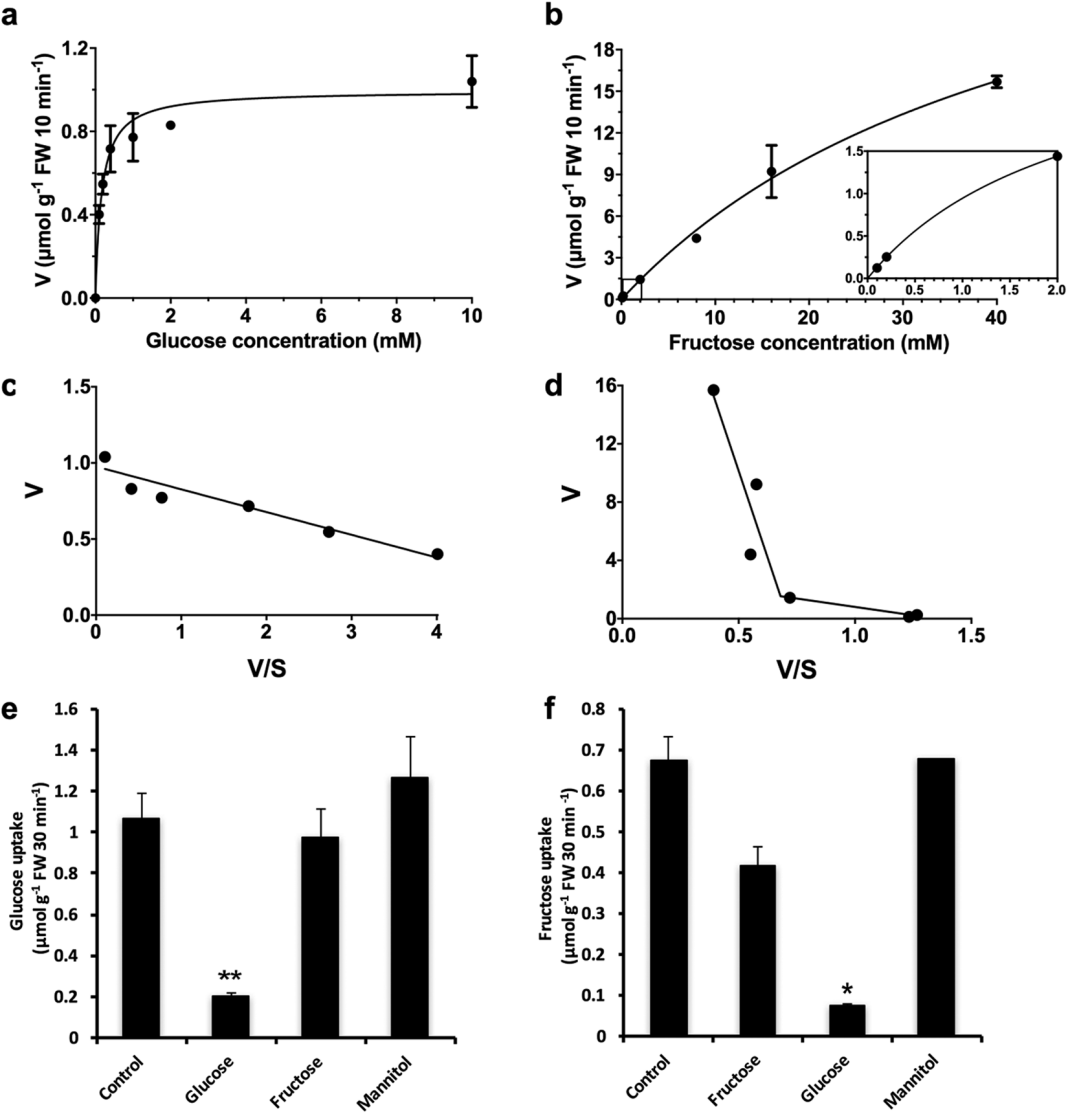
Kinetics of sugar uptake by *Arabidopsis* cells exposed to *B. cinerea* elicitation in the Millicell system. Concentration-dependent uptake rates of [^14^C]glucose (**a**) and [^14^C]fructose (**b**) in *Arabidopsis* cells collected 24 hours post-elicitation (mean + /−SE of at least 2 independent experiments). Insert: Concentration-dependent uptake rates of low [^14^C]fructose concentrations. The best fitting kinetics were determined using GraphPad Prism software. Eadie-Hofstee plots of the initial uptake rates of glucose (**c**) and fructose (**d**). The best fitting plots were determined using GraphPad Prism software. Competition of [^14^C]glucose (**e**) and [^14^C]fructose (**f**) uptake by ten-fold excess of glucose, fructose or mannitol in *Arabidopsis* cells elicited for 24 hours (mean + /− SE of at least 2 independent experiments). Asterisks represent significant differences compared to the control condition (Student’s *t*-test, * *P* < 0.05; ** *P* < 0.01).

We further investigated the kinetics of sugar uptake into 24 h-elicited cells which exhibited differences of uptake capacities for glucose and fructose. Mock-inoculated cells were not analysed because our study focused on the mechanisms of sugar acquisition by interacting cells. For glucose, initial uptake rate followed a monophasic Michaelis-Menten kinetic, indicating the involvement of a saturable transport system (Fig. 3a). We determined kinetic parameters by application of a computer-assisted non-linear regression analysis (GraphPad software): *K*_m_, 165 ± 27 μM glucose; *V*_max_, 1 ± 0.04 μmole^−1^ glucose 10 min^−1^ g FW^−1^. After the Eadie-Hofstee transformation, data points were fitted by linear regression suggesting the operation of only one high-affinity transport system for glucose (Fig. 3c). The saturation of fructose uptake never occurred, even for high concentrations of substrate (40 mM) (Fig. 3b), suggesting that fructose is taken up by a transport system with a low affinity compared to glucose. Accordingly, no *K*_m_ and *V*_max_ values could be calculated. The Eadie-Hofstee plots of the data were best fitted by two straight lines (Fig. 3d) which clearly indicates the involvement of two fructose transport systems with low and very low affinities. Then, the specificities of the monosaccharide carrier systems were tested with competing sugars. [^14^C]-Glucose uptake was not significantly affected by an excess of fructose (Fig. 3e). Therefore, fructose did not behave as a competitive inhibitor of glucose uptake. As shown in Fig. 3f, glucose was a strong competitive inhibitor of the [^14^C]-Fructose uptake, as we observed 90% of inhibition. The rate of fructose uptake was reduced by only 40% in the presence of an excess of fructose, confirming the low affinity for fructose. It suggests that glucose and fructose may use the same transporters but with different affinities.

Altogether, we demonstrated that both pathogen and host possess the capacity to compete for extracellular monosaccharides at the plant-fungus interface. Moreover, *Arabidopsis* cells elicited by *B. cinerea* activate a complex low and high affinity hexose uptake system.

### Elicitation by *B. cinerea* induces the expression of plant sugar transporter genes and the accumulation of the AtSTP13 protein

**Figure 4:**
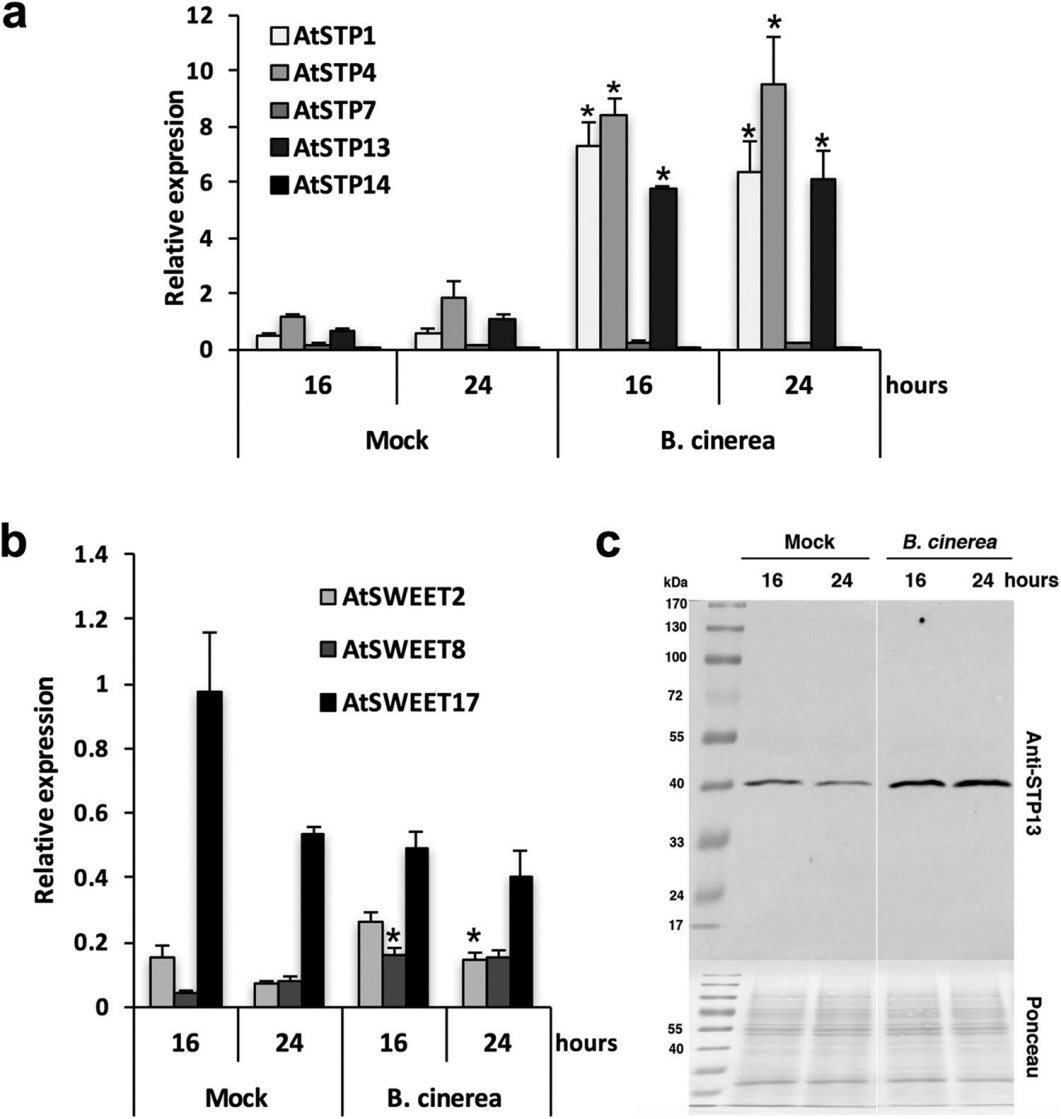
Expression of *Arabidopsis STP* and *SWEET* genes, and accumulation of the AtSTP13 protein in cells exposed to *B. cinerea* elicitation in the Millicell system. Relative expression of the 14 *STP* (**a**) and the 17 *SWEET* (**b**) genes in mock and *B. cinerea* treated cells. Genes that were expressed below the detection threshold are not presented. Gene expression analysis was performed by RT-qPCR and results were normalized to the plant reference gene *At4g26410*. Data represent mean (+/−SE) of at least 3 independent experiments. (**c**) Accumulation of the AtSTP13 protein in *Arabidopsis* cells exposed or not (mock) to *B. cinerea* in the Millicell system. Cells were collected at the indicated time points and proteins were extracted from microsomal fractions. Thirty micrograms of total microsomal proteins have been loaded in each lane. Protein immunoblot was performed with a polyclonal antibody raised against the AtSTP13 protein. Images belong to the same blot, with identical exposure parameters. Protein loading was visualized by staining blots with a Ponceau S solution. Asterisks represent significant differences compared to the corresponding mock condition (Student’s *t*-test, * *P* < 0.05).

Since glucose and fructose absorptions into *Arabidopsis* cells were enhanced by *B. cinerea* elicitation, we investigated whether sugar transporter genes belonging to both STP and SWEET families, which include 14 and 17 members, respectively^23,45^, were transcriptionally modulated. Only 5 *AtSTP* and 3 *AtSWEET* genes were expressed to a detectable level in *Arabidopsis* cultured cells. Transcript levels of *AtSTP7* and -14 were very low compared with other sugar transporter genes (Fig. 4a). Transcripts of 3 *AtSTP* genes, *AtSTP1*, -4 and -*13*, were relatively abundant in healthy non-elicited cells and were strongly induced (between 5 to 14 times) after elicitation (Fig. 4a). In comparison with the corresponding controls, the expression of *AtSWEET8* and *-2* was slightly stimulated in cells elicited for 16 and 24 hours, respectively (Fig. 4b). By contrast, the transcript level of *AtSWEET17*, encoding a tonoplastic facilitator^46^, declined moderately after 16 hours (Fig. 4b). Collectively, these data show that the increase of glucose and fructose uptakes into 16 and 24 h-elicited cells (Fig. 2b) is associated with the transcriptional up-regulation of several sugar transporter genes, *i.e. AtSTP1*, -4, -13, *AtSWEET8* and to a lesser extent *AtSWEET*2 (Fig. 4a,b).

In Lemonnier, *et al*.^27^, we produced a specific antibody raised against AtSTP13, which prompted us to examine the accumulation of this protein by western blot analysis. As shown in Fig. 4c, AtSTP13 was detected in healthy control cells and accumulated to a higher level in elicited cells. Because we previously showed that the amount of AtSTP13 correlates with the glucose uptake activity *in vivo* ^27^, we postulate that this sugar transporter may contribute substantially to the *Botrytis*-induced hexose absorption into *Arabidopsis* cells.

### The molecular dialogue with *B. cinerea* triggers major changes in host carbohydrate metabolism

**Figure 5:**
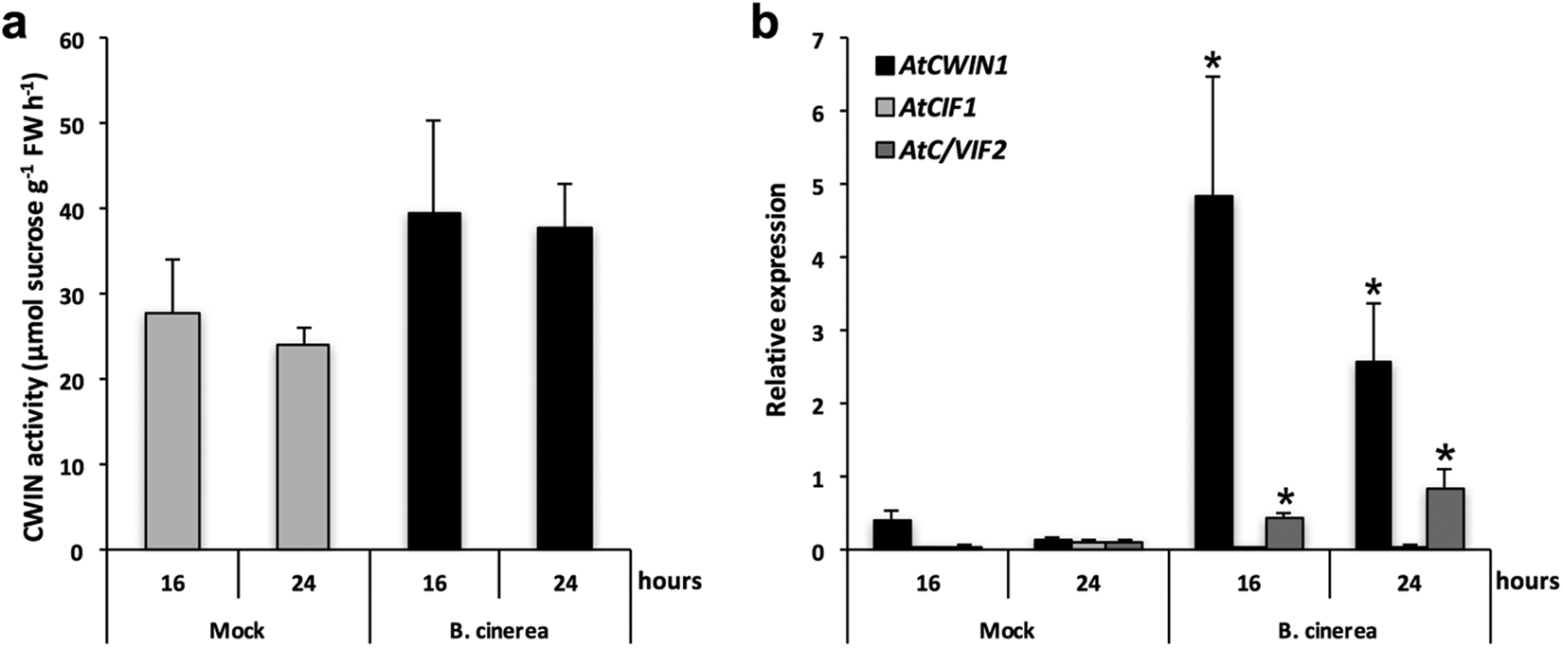
Cell wall invertase activity and expression of *Arabidopsis* invertase-related genes in cells exposed to *B. cinerea* elicitation in the Millicell system. (**a**) CWIN activity was assayed from insoluble extracts of *Arabidopsis* cells elicited or not (mock) with *B. cinerea*. Data represent mean (+/−SE) of 3 independent experiments. (**b**) Relative gene expression of *AtCWIN1*, *AtCIF1 and AtC/VIF2*. Gene expression analysis was performed by RT-qPCR and results were normalized to the plant reference gene *At4g26410*. Data represent mean (+/−SE) of at least 3 independent experiments. *AtCWIN2, -3, -4 and -5* are not presented because transcript levels were below the detection threshold. Asterisks represent significant differences compared to the corresponding mock condition (Student’s *t*-test, * *P* < 0.05).

We analysed the activity of cell wall invertases (CWINs) that would provide substrates to hexose–specific transporters. Since we supplied sucrose as a unique source of carbon, it was not surprising to detect a significant apoplastic sucrose degrading activity in healthy cells (Fig. 5). We found that the elicitation of *Arabidopsis* cells led to a slight stimulation in cell wall invertase activity that was increased by 42% and 56% at 16 and 24 hours post elicitation, respectively (Fig. 5a). Among the four *Arabidopsis AtCWIN* genes (*AtCWIN1*, *2*, 4 and 5) encoding functional sucrose cleaving enzymes^35^, only transcripts of *AtCWIN1* were detected in *Arabidopsis* cells showing a strong induction upon *Botrytis* challenge (Fig. 5b). Because this accumulation did not fully correlate with the low *Botrytis*-induced increase in CWIN activity (Fig. 5a), we monitored the expression of *AtCIF1* and *AtC/VIF2*, which encode specific invertase inhibitor proteins^47^. As shown in Fig. 5b, the level of *AtC/VIF2* transcripts strongly increased in elicited cells suggesting that the cell wall invertase activity is likely regulated at the transcriptional and post-translational level in this condition.

**Figure 6:**
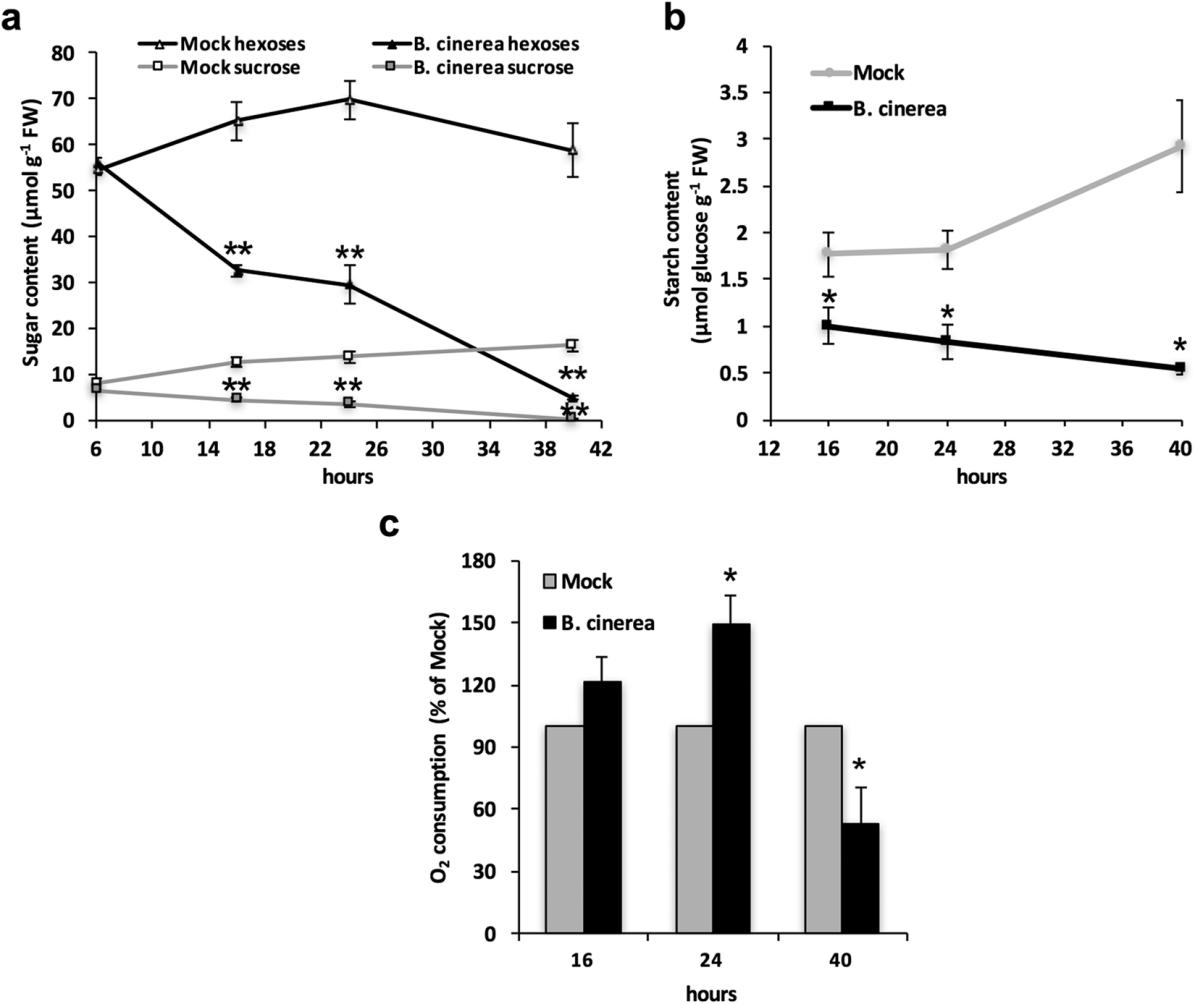
Sugar content and oxygen consumption in *Arabidopis* cells exposed to *B. cinerea* in the Millicell system. Time-course analysis of soluble sugars (**a**) and starch (**b**) contents in mock and *Botrytis-*challenged *Arabidopsis* cells. The level of soluble hexoses corresponds to the total level of soluble glucose and fructose. The amounts of soluble and insoluble sugars were assessed using enzymatic assays. Data represent mean (+/−SE) of at least 3 independent experiments. (**c**) Rate of oxygen consumption was measured in mock and *Botrytis* challenged cells using a Clark electrodes. The value of the corresponding mock condition was set to 100% for each time point. Results represent mean (+/−SE) of 3 independent experiments. Asterisks represent significant differences compared to the corresponding mock condition (Student’s *t*-test, * *P* < 0.05; ** *P* < 0.01).

We further investigated whether the increase in hexose uptake was reflected by changes in host carbohydrate metabolism. The concentrations of the major sugars involved in metabolism, *i.e*. glucose, fructose and sucrose, as well as starch, were measured from total extracts of host cells. As seen in Fig. 6a, the level of intracellular soluble hexoses (glucose + fructose) was considerably higher (about 10 times) than sucrose, the content of those sugars being relatively stable over time in mock-treated cells. At the early stage of the elicitation, no major change was observed in the sugar content (Fig. 6a). By contrast, a dramatic drop in hexose and sucrose concentrations occurred from 16 hours post-elicitation, reaching 45 and 26% of the control values for respective sugars at 24 hours post-elicitation (Fig. 6a). After 40 hours, we were able to measure only weak levels of soluble sugars. The amount of starch, which was very low in heterotrophic cultured cells, was strongly reduced after 24 and 40 hours of elicitation (Fig. 6b).

Globally, we showed that elicitation resulted in a decrease of the soluble and insoluble sugar contents in *Arabidopsis* cells. The content of intracellular sugars is a balance between the production of carbohydrates through photosynthesis and consumption *via* the cellular respiration. To evaluate the contribution of the host primary metabolism in this process, we monitored the oxygen consumption of the elicited cell suspensions using Clark electrodes. Interestingly, after 16 and 24 hours post-elicitation, O_2_ consumption was increased by 20 and 50% respectively, indicating that the metabolism of the host was stimulated upon elicitation (Fig. 6c). After 40 hours, the uptake of O_2_ was strongly altered, further supporting to the assumption that cells are dying at this stage of the interaction (Fig. 6c).

## Discussion

Here, we present an innovative system to study the molecular communication between plant and pathogen cells. Using Millicell, we were able to set up a molecular dialogue between an *Arabidopsis* cell suspension and the necrotrophic fungus *B. cinerea*, without any physical contact. The experimental set-up described here allows the free diffusion of secreted molecules produced by both partners and opens the opportunity to study temporal responses from either the host or the fungus. To our knowledge, the use of this system has never been reported for the study of the interactions between plants and pathogens.

The molecular basis of the dialogue between *B. cinerea* and its host has been extensively studied with the aim to search fungal virulence factors^11,48^. *Botrytis* secretome includes a large array of molecules with a variety of activities. Most of the secreted proteins are involved in the degradation of host cellular barriers or show necrotizing activities^10,12^. In some cases, they are recognized as PAMPs or release DAMPs triggering plant immune responses^49,50^. For example, the endopolygalacturonase BcPG1, which is required for the full virulence of *B. cinerea*, is perceived as PAMPs but also degrades host cell wall pectin and release oligogalacturonides (OGs)^51,52^. OGs are recognized by the RLK Wall Associated Kinase 1 (WAK1) and trigger a variety of PAMP responses, such as the induction of the camalexin biosynthesis gene *PAD3* ^14,49,53^. Transcriptional changes of several pathogen-responsive genes exhibited by elicited host cells indicate that the Millicell is a valid system to elicit host defence responses. The molecular communication occurring in the Millicell likely involves the perception of danger signals from *B. cinerea*. The stop of cell proliferation and eventually the host cell death are likely due to the activity of toxins or hydrolytic enzymes produced by *B. cinerea* and/or the activation of the HR-like cell death.

**Figure 7:**
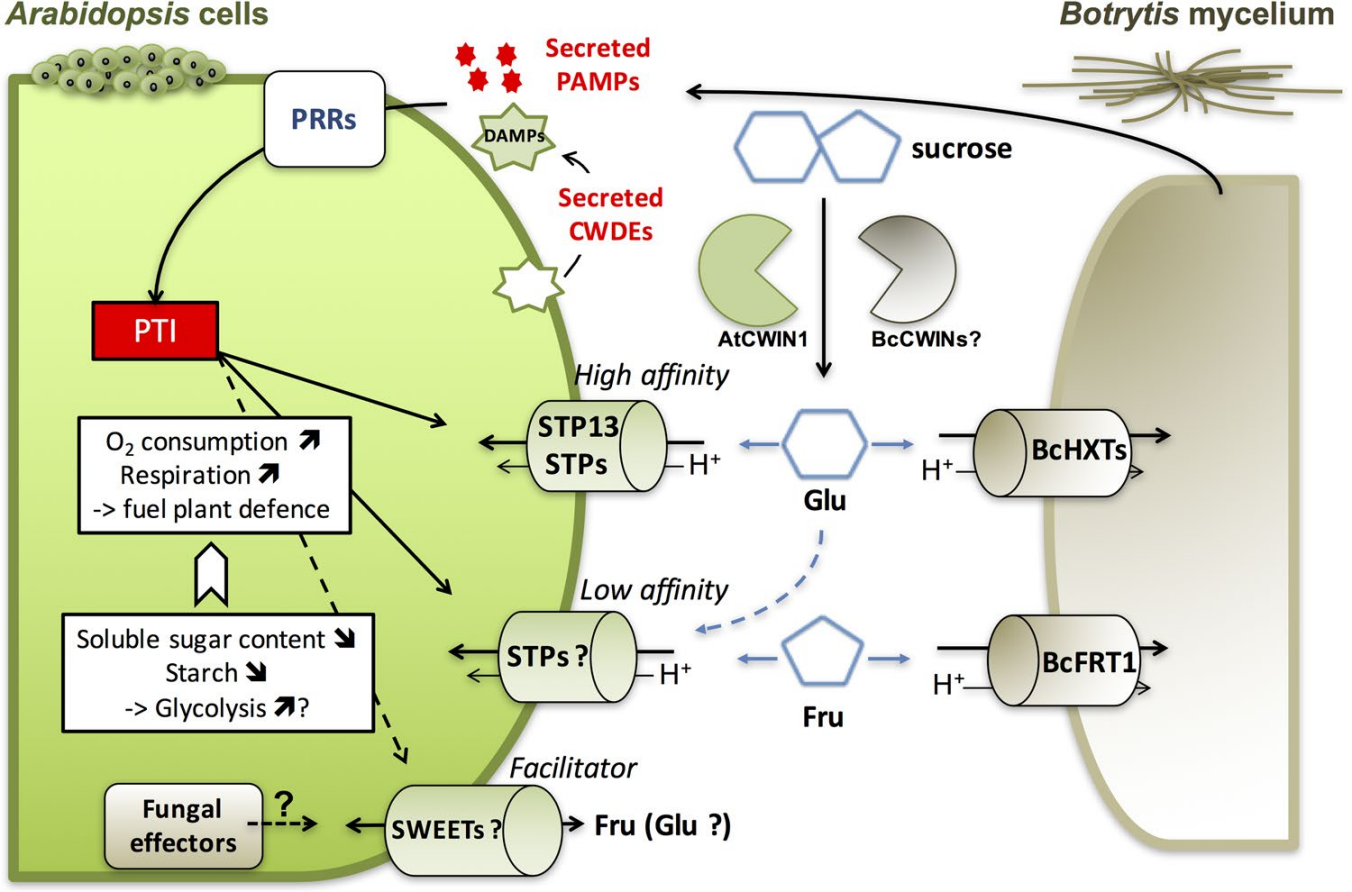
Model of the sugar competition between *Arabidopsis* cells and *Botrytis cinerea*. Upon infection, secreted PAMPs and/or host-derived signals (DAMPs) are recognized by PRRs, resulting in a transcriptional reprogramming of host sugar transporters. Putative fungal effectors may also manipulate host SWEET gene expression. Apoplastic sucrose is cleaved into glucose and fructose by cell wall invertases which can originate from both the host (AtCWIN1) and/or the pathogen (BcCWINs). *Arabidopsis* and *Botrytis* compete for free hexoses through specific activities of sugar transporters (BcHXTs and BcFRT1 for *B. cinerea*) which have differential specificity and affinity for hexoses. On the plant side, the *Botrytis*-induced sugar uptake activity is accompanied with a reduction of internal sugars and an increased O_2_ consumption suggesting an enhanced activity of the glycolysis and the cellular respiration. Together, these changes in host primary metabolism may contribute to deprive the pathogen from sugar resources and to fuel plant defence responses.

The distinct compartments of the Millicell gives the opportunity to handle separately elicited host and fungal cells with the aim to explore the competition for sugars at the plant/pathogen interface. Information concerning carbon uptake by *B. cinerea* is fragmentary. In Doehlemann, *et al*.^37^, fructose and glucose uptake into conidia appeared to be mediated by low-specificity hexose transporters with higher affinity to glucose than fructose. *In vivo* NMR analysis of sunflower cotyledons infected by *B. cinerea* revealed that hexoses imported from the plant are converted into mannitol^38^. Here, we addressed the hexose uptake from growing mycelium during pathogenesis. We were able to measure significant hexose uptake with similar rates for glucose and fructose. We further demonstrated that hexoses are actively taken up into *Botrytis* mycelium through a mechanism involving the proton motive force. This result is supported by studies showing that sugar acquisition by *Botrytis* is based on a multigenic hexose uptake system involving the fructose-specific transporter BcFRT1 and several putative hexose transporters (BcHXTs)^35,37,38^ (Fig. 7). Additional work will be necessary to determine individual roles of *Botrytis* hexose transporters during the different stages of pathogenesis.

Few studies have investigated the carbon uptake by plant cells challenged with necrotrophic pathogens. Such *in vivo* analyses have to cope with technical difficulties, *i.e*. death of host cells and the concurrent fungal absorption of sugars. In a previous study, we have reported the induction of the Sugar Transporter Protein AtSTP13 in *Arabidopsis* leaves challenged with *B. cinerea* ^27^. However, the specific measurement of the host sugar absorption was not achievable in infected leaves since host and fungal tissues were closely associated, and plant tissues dramatically macerated. Azevedo, *et al*.^54^ reported that pine cell suspension co-cultured with *Botrytis* conidia displayed an enhanced glucose transport. However, the concomitant presence of host and fungal cells makes the findings difficult to interpret since it has been demonstrated that *B. cinerea* is competent to absorb extracellular sugars (this work; Doehlemann, *et al*.^37^) and should contribute to the overall glucose uptake in such experimental conditions. Here, we were able to separate host responses from the ones of *Botrytis* using the Millicell system and provide evidence of the activation of a complex sugar transport system in *Botrytis*-challenged cells (depicted in Fig. 7). We demonstrated that glucose and fructose are predominantly taken up through a proton-driven transport system, which exhibits a high and low affinity for glucose and fructose, respectively. We determined kinetic properties, which are compatible with the involvement of high-affinity monosaccharide/H^+^ transporters belonging to the STP family^45^. We also highlighted a very low-affinity component for fructose, which may involve facilitator proteins, such as SWEETs, that drive the pH-independent diffusion of sugars across cell membranes down a concentration gradient^23^. Accordingly, a transcriptional screening of sugar transporter genes belonging to the AtSTP and AtSWEET families was performed during the course of the elicitation. We found that several sugar transporter-encoding genes, *i.e. AtSTP1*, -4, -13, and *AtSWEET2*, -*8*, were induced upon elicitation. The expression of *AtSTP13* was previously shown to be up-regulated by *B. cinerea* and *Pseudomonas syringae* leaf infections or upon PAMP treatment^27,29,55^. *AtSTP4* transcripts accumulated to high level in *Arabidopsis* suspension cells exposed to chitin^56^, which is a major component of fungal cell walls recognized as PAMPs by the LysM receptor kinase CERK1 (Chitin Elicitors Receptor Kinase 1)^57^. In leaf tissues infected by the fungal biotroph *Erysiphe cichoracearum*, the increased glucose uptake was accompanied by the induction of *AtSTP4* expression^34^. *P. syringae* highly induced the mRNA level of *AtSWEET8* in *Arabidopsis* leaves^23^. The expression of *AtSWEET2* in root cells was induced by the soil-born oomycete *Pythium* ^58^. The loss-of-function mutant *sweet2* was more susceptible to this pathogen, suggesting a role of the tonoplastic AtSWEET2 in limiting the availability of sugars in the rhizosphere^58^. Interestingly, these genes, with the exception of *AtSWEET2*, were previously found to encode plasma membrane-localised proteins that transport glucose or fructose in heterologous systems or *in vivo* ^23,45,58,59^ indicating that each may contribute at different levels to the overall hexose uptake activity into elicited cells.

Different roles may be hypothesized for the *Botrytis*-induced STP and SWEET sugar transporters (Fig. 7). The induction of SWEETs would contribute negatively to the sugar uptake and promote pathogen growth by increasing sugar efflux from the cytosol into the apoplast^19,23–25^. The deficiency in *AtSWEET4* led to a better tolerance to *B. cinerea* ^60^ and the overexpressing line showed an increased susceptibility to the non-host bacteria *P. s*. pv *phaseolicola* ^61^. We may speculate that *B. cinerea* manipulates host SWEET efflux machinery via the secretion of effectors. The millicell may be an appropriate system to find such putative effectors. By contrast, the role of STPs, which are all characterized as proton/sugar symporters of the plasma membrane, would be to actively retrieve hexoses from the apoplast and consequently favour host defence. In agreement, STP13-deficient and overexpressing lines exhibited enhanced susceptibility and increased resistance to *B. cinerea*, respectively^27^. The double mutation *stp1 stp13* leads to an increased concentration of hexoses in the leaf apoplast upon flg22 elicitation and an enhanced proliferation of the bacteria *P. syringae* ^29^. AtSTP13 is associated with PRR complexes (FLS2 and/or BAK1) and phosphorylation-dependent activation of AtSTP13 activity is required for antibacterial defence. According to Yamada, *et al*.^29^, the function of AtSTP13 would be to deprive the pathogen of carbon resource by remobilizing apoplastic hexoses and to suppress bacterial virulence by reducing the sugar-mediated activation of T3SS effector delivery.

The importance of cell wall invertases (CWIN) in the release of free hexoses in the apoplast has been reported in many plant-pathogen interactions^33^. Our previous report showed that AtCWIN1 is the main contributor to the *Botrytis*-induced CWIN activity^35^. Here, we also provide evidence that it is also probably subjected to a posttranslational regulation through the suppressing activity of invertase inhibitor, such as AtC/VIF2.

Finally, we explored the roles of imported hexoses into host cells. By contrast with the enhanced hexose absorption into host cells, we showed that the amount of the main soluble sugars and starch dramatically decreased. Similar results have been described in tomato leaves infected with *B. cinerea* ^62^. The authors suggested that such reduction was attributed to the down regulation of photosynthesis. In the Millicell, cells were grown in heterotrophic situation with external sucrose as the sole carbon source, thus the contribution of photosynthesis to sugar production is likely negligible. Therefore, we postulate that the low sugar content is mainly due to an increase of the consumption of carbohydrates by defending cells to produce energy (Fig. 7). It is assumed that plant defence responses are associated with an increased demand for energy provided by primary metabolic pathways^30^. Carbohydrates are the main sources for the cellular respiration that generates energy equivalents and carbon skeletons used in the biosynthesis of various metabolites^31^. Hence, we examined the oxygen uptake of *Arabidopsis* cells elicited in the Millicell, which is indicative of the intensity of the cellular respiration because cells were grown in heterotrophic conditions. We demonstrated that the cellular respiration is strongly stimulated in elicited cells until they become non-metabolically active. We make the conclusion that sugars are used to fuel plant defence by increasing the activity of respiratory metabolism (Fig. 7). This cellular process can be divided into three main pathways, glycolysis, the mitochondrial tricarboxylic acid (TCA) cycle and mitochondrial electron transport^63^. Further work will be necessary to determine how each pathway contributes to this increased metabolic demand by defending cells.

To conclude, this work shows that the molecular dialogue between *Arabidopsis* cells and *B. cinerea* triggers major changes in host carbon metabolism, including enhanced apoplastic sucrose degradation, hexose uptake, carbohydrate consumption and cellular respiration, which may contribute to deprive the pathogen from resources and fuel plant defence responses.

## Methods

### Biological material and growth conditions

*Arabidopsis thaliana* cell suspension culture (ecotype Col-0) was grown in Gamborg B5 medium (Duchefa Biochemie) supplemented with sucrose (2% w/v), α-naphthaleneacetic acid (1 μM) and 6-benzylaminopurine (4.44 μM), with pH adjusted to 5.8. The cell suspension culture has been initiated from leaf tissues. Cells were maintained by subculturing every 7 days 10 ml of saturated culture into 40 ml of fresh media. Cells were grown under a 16 h (light)/8 h (dark) photoperiod and rotated at 140 rpm on an orbital shaker at a temperature of 22 °C.

*Botrytis cinerea* strain B05.10^64^ was grown on Difco potato dextrose agar (Becton-Dickinson) under a 16 h (light)/8 h (dark) photoperiod at a temperature of 22 °C. Conidia were harvested in sterile water and filtered through miracloth (EMD Chemicals).

### Establishment of the interaction between *A. thaliana* cells with *B. cinerea* in the Millicell system

Hydrophilic PTFE cell culture inserts (0.4 μm diameter pores, 30 mm diameter, Merck Millipore Ltd) were placed in wells of 6-well culture plates. *Arabidopsis* cell suspension was cultured for 4 days to reach the exponential phase of growth. Twenty milliliters of *Arabidopsis* cells were washed and resuspended in the same volume of fresh supplemented Gamborg B5 medium. At time 0, a volume of 1.5 ml of *Arabidopsis* cells was placed in the apical side of the millicell and 30,000 conidia of *B. cinerea* were added to 1.5 ml supplemented Gamborg B5 medium in the basolateral compartment. In the mock condition, conidia were omitted. Culture plates were incubated at 22 °C under a 16 h (light)/8 h (dark) photoperiod with tridimensional shaking (50 rpm) and cells were collected by vacuum filtration at the indicated time points.

For neutral red staining, one volume of *Arabidopsis* cells was incubated with half volume of neutral red solution (0.1%) for 2 min and observed under light microscope.

### Glucose and fructose uptake into *Arabidopsis* and *B. cinerea* cells

*Arabidopsis* cells or *Botrytis* mycelium were collected, washed and incubated for 45 min in equilibration buffer (Gamborg B5 medium, 20 mM MES-KOH pH 5.8) under agitation. *Arabidopsis* cells were resuspended at a final concentration of 20 mg ml^−1^ FW. Sugar uptake assays were performed as previously described in Veillet, *et al*.^35^. Briefly, samples were incubated in equilibration buffer containing glucose or fructose (0.2 mM) and D-[U-^14^C]-sugars (glucose or fructose; 0.1 μCi ml^−1^) under agitation. Incubation times are specified in figure legends. To evaluate the involvement of proton-motive force in sugar uptake, carbonyl cyanide m-chlorophenylhydrazone (20 μM) was added in the incubation buffer 10 min before addition of radiolabelled sugars. After incubation, *Arabidopsis* cells and *B. cinerea* mycelium were washed three times with equilibration medium and collected by filtration. Samples were left overnight in digestion buffer (36.4% perchloric acid, 0.017% triton X-100 and 8.1% hydrogen peroxide w/v) at 60 °C. Incorporated radioactivity was determined by liquid scintillation counting (Tri-Carb 2910 PR, PerkinElmer).

For the determination of kinetic parameters of sugar uptake, *Arabidopsis* cells collected 24 hours post-elicitation were incubated for 10 min with a substrate concentration range of 0.1–10 mM for glucose and 0.1–40 mM for fructose. The transport kinetic parameters were determined after a computer-assisted non-linear regression analysis of the experimental initial uptake rates of labelled glucose and fructose (GraphPad prism software).

For the determination of substrate specificities, inhibition of glucose and fructose transport was assessed by adding simultaneously competitive unlabelled sugars (2 mM for glucose, fructose or mannitol) together with radiolabeled sugars (0.2 mM) in the incubation buffer.

### RNA extraction and quantitative reverse transcription-PCR (qRT-PCR) analysis

Total RNA was extracted from frozen ground *Arabidopsis* cells using the Spectrum Plant Total RNA Kit (Sigma-Aldrich) according to the manufacturer’s instructions. DNAse I treatment, reverse transcription, and real-time quantitative RT-PCR were performed as described in Veillet, *et al*.^35^. Target gene expression was normalized to the expression of the plant gene *At4g26410*, previously described as a stable reference gene^65^. Results were expressed as relative gene expression according to the 2^−ΔCt^ method described by Schmittgen and Livak^66^. Primers have been designed using Primer3^67^ in conjunction with Netprimer (www.premierbiosoft.com/netprimer) and tested for their specificity and efficiency (≥90%). Sequences of the primers used in this study are listed in Supplementary Table 2.

### Soluble and insoluble sugar quantification

Frozen ground *Arabidopsis* cells (25 mg) were serially extracted (3 times) in methanol/chloroform/water (60/25/15, v/v/v). The mixture was centrifuged at 1,200 g for 10 min at 20 °C. Supernatants were pooled (3.3 ml) and mixed with 1.8 ml of water and centrifuged at 1,200 g for 15 min at 20 °C. The supernatant was collected, evaporated in a centrifugal vacuum evaporator (MiVac QUATTRO concentrator) at 50 °C for 3 hours and the pellet was resuspended in water. The soluble glucose, fructose and sucrose content of sample extracts was measured using the Sucrose/D-Fructose/D-Glucose Assay Kit (Megazyme) according to the manufacturer’s instructions. Starch content was quantified from the pellet obtained after methanol/chloroform/water extraction using the Total Starch HK Assay Kit (Megazyme) according to the manufacturer’s instructions.

### Determination of cell wall invertase activity

Total extracts were made by mixing frozen ground *Arabidopsis* cells (50 mg) with 300 μl of ice-cold extraction buffer (50 mM HEPES pH7.5, 5 mM EDTA, 5 mM DTT and 1 mM PMSF). Protocols for the isolation of the insoluble fraction and the measurement of the cell wall invertase activity were described in Veillet, *et al*.^35^.

### Determination of cell viability

The Cell Titer 96 Aqueous One solution cell proliferation assay (Promega) was used to quantify relative cell viability. Experiments were performed according to the manufacturer’s instructions. This colorimetric assay measures the reduction of MTS into formazan by metabolically active cells.

### Oxygen uptake measurement

Growing *Arabidopsis* cells were vacuum filtered and resuspended at a concentration of 100 mg ml^−1^ in fresh Gamborg B5 medium supplemented with sucrose (2%), α-naphthaleneacetic acid (1 μM) and 6-benzylaminopurine (4.44 μM) at pH 5.8. The consumption of dissolved oxygen by *Arabidopsis* suspension cells (20 mg FW) was monitored for 20 min using Clark electrodes (Dual digital model 20, rank brothers ltd).

### Western blot analysis

A microsomal fraction was prepared from 500 mg of frozen ground *Arabidopsis* suspension cells as described in Lemonnier, *et al*.^27^. Thirty microgrammes of total microsomal proteins were separated by SDS-PAGE (10% acrylamide) and blotted on nitrocellulose membrane (Hybond ECL, GE Healthcare) for western blot analysis. Blots were stained with a Ponceau S solution (Sigma-Aldrich) to visualize protein loading. Immunodetection of the AtSTP13 protein was assessed by incubating blots with a purified AtSTP13 antiserum described in Lemonnier, *et al*.^27^. Detection was realized using ECL Prime Western Blotting Detection Reagent (GE Healthcare) and imaged on LAS-3000 Imaging System (Fuji).

### Statistical and computer-assisted analysis

Statistical and computer-assisted analyses were performed using the GraphPad Prism version 7.00 for Mac, GraphPad Software, La Jolla California USA, www.graphpad.com.

## Electronic supplementary material

Supplementary information
