# Supplementary material for: The molecular dialogue between Arabidopsis thaliana and the necrotrophic fungus Botrytis cinerea leads to major changes in host carbon metabolism
Source: Sci Rep. 2017 Dec 7;7:17121. doi: 10.1038/s41598-017-17413-y (PMC5719352; doi:10.1038/s41598-017-17413-y)
Supplement: Supplementary file 1 — Supplementary information [file 41598_2017_17413_MOESM1_ESM.pdf]

The molecular dialogue between *Arabidopsis thaliana* and the necrotrophic fungus *Botrytis cinerea* leads to major changes in host carbon metabolism

Florian Veillet[1], Cécile Gaillard[1], Pauline Lemonnier[1,2], Pierre Coutos-Thévenot[1] and Sylvain La Camera[1]*

[1] Laboratoire Ecologie et Biologie des Interactions, Equipe "SEVE-Sucres et Echanges Végétaux-Environnement", Université de Poitiers, UMR CNRS 7267, F-86073 POITIERS, France.

[2] present address: Department of Plant Biology and Institute for Genomic Biology, University of Illinois at Urbana-Champaign, Urbana, Illinois 61801, USA.

*** Correspondence :**

Sylvain La Camera

sylvain.la.camera@univ-poitiers.fr

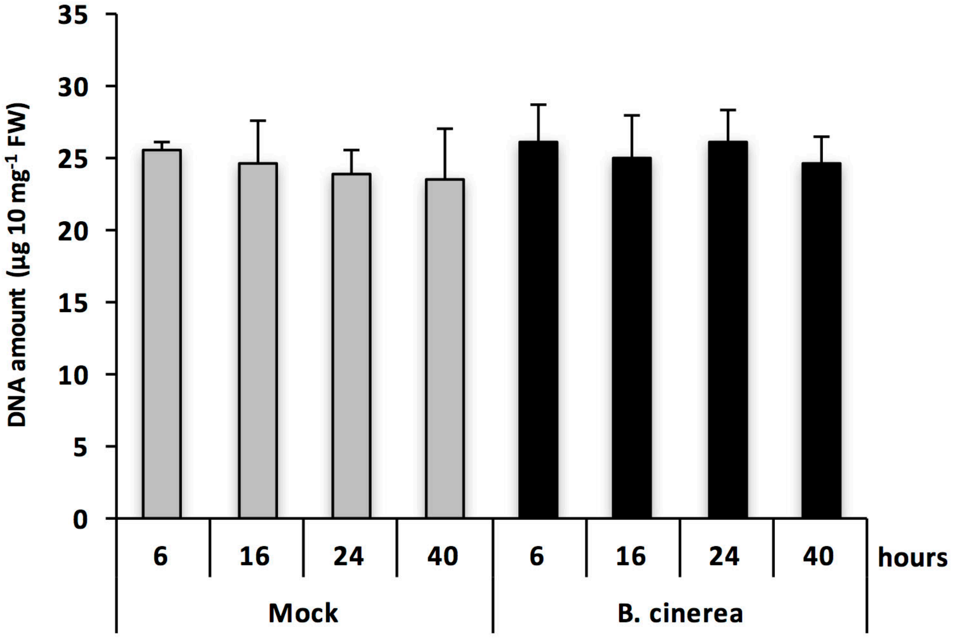

Supplementary figure 1 : Absolute DNA content by quantitative PCR in *Arabidopsis* cells exposed to *B. cinerea* in the Millicell system. The amount of genomic DNA was determined using the standard curve method according to Gachon and Saindrenan (2004). The absolute quantification of DNA copy number was determined after PCR amplification of the plant gene *AtiASK* gene from genomic DNA extracted from 10 mg of *Arabidopsis* cells (fresh weight). Data represent mean (+/-SE) of at least 2 independent experiments.

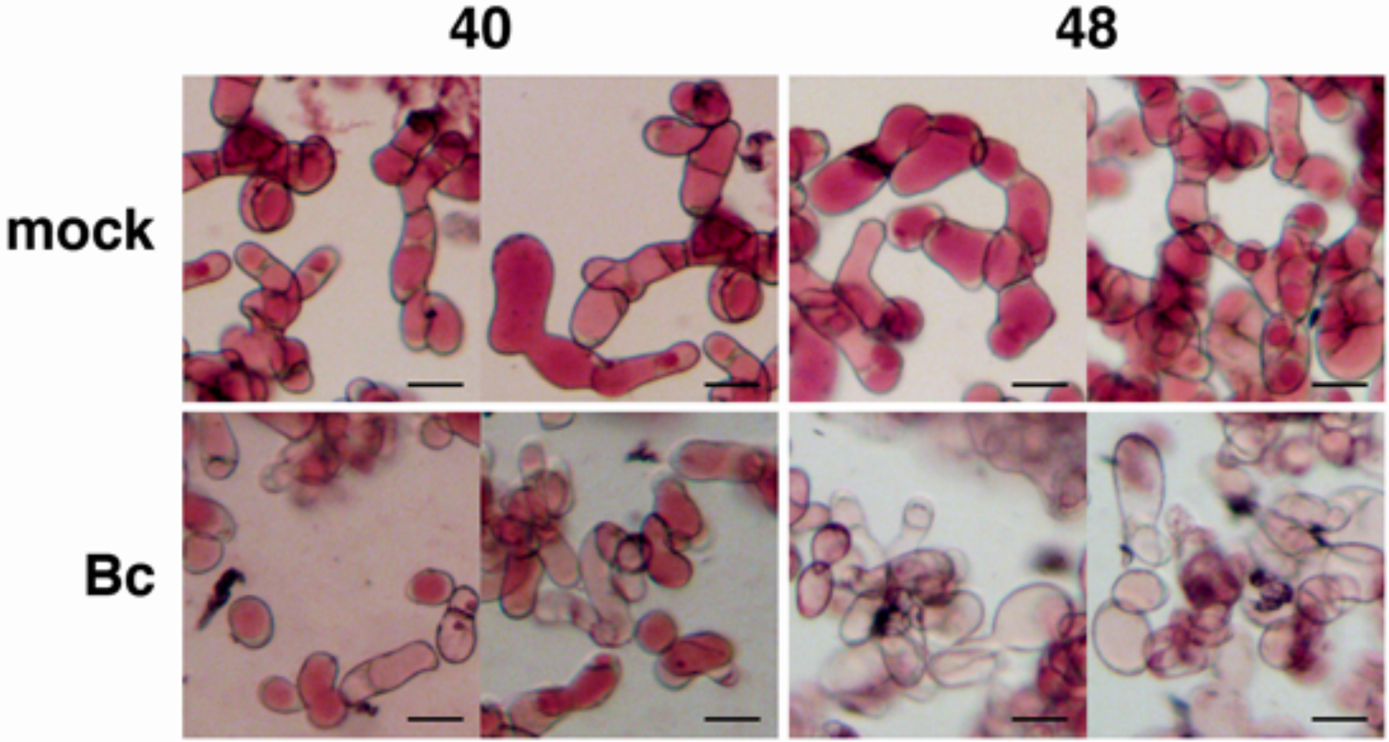

Supplementary figure 2 : Neutral red staining of mock- and *B. cinerea*- treated *Arabidopsis* cells (c) in the Millicell. Neutral red staining were made after 40 and 48 hours. One volume of *Arabidopsis* cells was incubated with half volume of neutral red solution (0.1%) for 2 min and observed under light microscope. The staining procedure and the parameters of optical acquisition are identical for each condition. Scale bar = 25 μm. Bc : *Botrytis cinerea*

Supplementary table 1 : Uptake of [^{14}C]-labeled hexoses into *Arabidopsis* cells exposed to *B. cinerea* in the Millicell system. Active uptake (µmole g^{-1} FW 30 min^{-1}) results from the difference between total uptake and CCCP-insensitive uptake. The inhibition of uptake by CCCP is relative to the total uptake of [^{14}C]-labeled hexoses into *Arabidopsis* cells. Data represent mean (+/-SE) of at least 3 independent experiments.

	Hours	Active uptake ± SE	Inhibition of uptake by CCCP (%)
Glucose			
Mock	16	0.57 ± 0.08	87.41
	24	0.49 ± 0.08	82.47
	40	0.53 ± 0.14	85.63
B. cinerea	16	1.20 ± 0.11	88.56
	24	1.34 ± 0.17	88.30
	40	0.03 ± 0.02	34.98
Fructose			
Mock	16	0.17 ± 0.02	87.72
	24	0.19 ± 0.03	88.31
	40	0.14 ± 0.04	89.68
B. cinerea	16	0.46 ± 0.04	89.18
	24	0.45 ± 0.04	90.82
	40	0.004 ± 0.001	15.75

Supplementary table 2 : Primers used in this study for gene expression and DNAquantification analyzes.

Genes	Loci	Forward primers (F)	Reverse primers (R)	References
Reference (REF)	*At4g26410*	GAGCTGAAGTGGCTTCCATGAC	GGTCCGACATACCCATGATCC	Czechowski *et al.*, 2005
AtSTP1	*At1g11260*	ATGCCGTCTTTCCTCAAGCG	TAGCGTCGGGCTATCGTACT	Yamada *et al.*, 2011
AtSTP2	*At1g07340*	GCTGTGTCATTGCTGCTGTT	TCGTACACGTGTGGGAAAAA	Yamada *et al.*, 2011
AtSTP3	*At5g61520*	GTAACTTGCGACACGAGCATCT	ACCGTTGGGTTTGGAGAGAGT	Lemonnier *et al.*, 2014
AtSTP4	*At3g19930*	CTTCTCTCTTTGCCTCCACCAT	CGACTCCGAAACCAAGTAGGA	Lemonnier *et al.*, 2014
AtSTP5	*At1g34580*	GGGGACGGAGAGATGAAGAAG	CTTGAAATCGCAGAGCGTTG	Lemonnier *et al.*, 2014
AtSTP6	*At3g05960*	GCACTCGTGGCCAGTTTCGTC	TGAGACCGACACCAATCAAG	Yamada *et al.*, 2011
AtSTP7	*At4g02050*	ACTTAGCTATGCTTCTTGCC	ATGAGTTGGTGCCACTTCTG	Yamada *et al.*, 2011
AtSTP8	*At5g26250*	GGCTAGCTTCTTTGCTTCGGCT	AGATGGAAGCGAGTTGCATT	Yamada *et al.*, 2011
AtSTP9	*At1g50310*	TGTGAGGCTGCTAAGAAAGTGG	TAAAGAGAACGGGGGCGTAGA	Lemonnier *et al.*, 2014
AtSTP10	*At3g19940*	CGGTAGACTGTTGCTTGGTGTT	GCTCCTCTTATCTTCGCTGGA	Lemonnier *et al.*, 2014
AtSTP11	*At5g23270*	CTTTCCTTGCCTCCACCATTAC	AATCCGACACCTACACCGAGA	Lemonnier *et al.*, 2014
AtSTP12	*At4g21480*	GACAATGGATTCGTTTCAGC	GAGAAACGCTGTCAAATCTGC	Yamada *et al.*, 2011
AtSTP13	*At5g26340*	TATGGGACCGCCAAGATTAAA	AAGCTCCGACCGTTAGAAGAA	Lemonnier *et al.*, 2014
AtSTP14	*At1g77210*	ACTCATGTTCCTCGGTGGAC	ACTTTGATCAGCACGGCTTT	Yamada *et al.*, 2011
AtSWEET1	*At1g21460*	TCGTCTATGGTCTAATCGGTCGT	CCCTAATGCACATCCAAACCCA	This work
AtSWEET2	*At3g14770*	CACGGTGGTACTTTGTCGGG	AACGGCATAAACTCAACGCTCT	This work
AtSWEET3	*At5g53190*	GAGTCGGCATCCTTCTCGAA	ACCAAGGCTGAGATTGCTGTC	This work
AtSWEET4	*At3g28007*	TGCCATTCTCTCTCTCACTTGC	ACTGCTCCTGATACCGTTCCA	This work
AtSWEET5	*At5g62850*	TCTTCGCTACCTCCCCTGTC	TGTGCAGAAGATCACCACCG	This work
AtSWEET6	*At1g66770*	GGTTACGTTGGTGAAGTCGGA	CTCGGGTTCAGTTTGAGCGA	This work
AtSWEET7	*At4g10850*	ACGTCGGTTTATCAAGTGCGA	TCTTGGTTGGTATTCGCGGT	This work
AtSWEET8	*At5g40260*	GCCATTCTTGTTGTCTTTGGTCT	CTTCACCGTCTTCTCCTTTGGA	This work
AtSWEET9	*At2g39060*	ACGCCGTCATGTGGTTCTTT	TGCTAGTTGGTTCTCTGTTGGC	This work
AtSWEET10	*At5g50790*	AACTCCTTTGCCTTTGTCGTACA	GTCAACACGAAGATTGCGCC	This work
AtSWEET11	*At3g48740*	TCCTTCTCCTAACAACTTATATACCATG	TCCTATAGAACGTTGGCACAGGA	Chen *et al.*, 2010
AtSWEET12	*At5g23660*	AAAGCTGATATCTTCTTACTACTTCGAA	CTTACAAATCCTATAGAACGTTGGCAC	Chen *et al.*, 2010
AtSWEET13	*At5g50800*	GCTAACGACCGTCATACCCG	GAAGAGCAGGCGGTTGATGA	This work
AtSWEET14	*At4g25010*	AAACGCTGTGGGATGCTTCA	TTCAAGAGCCCAAGAACCTTCA	This work
AtSWEET15	*At5g13170*	TGAAAACGGCAAGTGCTACG	CCTGACGAGCCGAGAGAAAG	This work
AtSWEET16	*At3g16690*	TGCTGAATCCATCTACGTTCTCA	ACAGAGTTCTTGTTCCCGCAA	This work
AtSWEET17	*At4g15920*	AGTGACAACAAAGAGCGTGAAATAC	ACTTAAACCGTTGCTTAAACCAACC	Chen *et al.*, 2010
AtCWIN1	*At3g13790*	CGGAAGTGGAATGTGGGAAT	TGAAACCATTGTCCGGTACG	Veillet *et al.*, 2016
AtCWIN2	*At3g52600*	ACATGGTCCGGTTCAGCTAC	CTTGAGGTATGGGTCGGAAA	Veillet *et al.*, 2016
AtCWIN4	*At2g36190*	CAATCGTACCGGGAAAAGGA	CCGTCTTTGGAGAACCAAGC	Veillet *et al.*, 2016
AtCWIN5	*At3g13784*	GGGTCGGTAATGGAGGTTCA	AGCCCGGTTCAATCACATCT	Veillet *et al.*, 2016
AtCIF1	*At1g47960*	ACACTTCTGGCCTCGCTCTC	AATGGCTTCGGGAACATCAG	Veillet *et al.*, 2016
AtC/VIF2	*At5g64620*	TCTCATCTTCCTCCTCCTCGTT	ACCTTTTGTGTCGGCTGTGG	Veillet *et al.*, 2016
AtPAD3	*At3g26830*	TGCTCCCAAGACAGACAATG	GTTTTGGATCACGACCCATC	La Camera *et al.*, 2011
AtPR4	*At3g04720*	GTTTAAGGGTGAAGAACACAAGAAC	ATTGAACATTGCTACATCCAAATC	Cabello and and Chan, 2012
AtPLP2	*At2g26560*	GTAGCTGGTTGGGGACTATTGA	CGGTAGCGATATCAACAGAAGC	La Camera *et al.*, 2005
AtGRXS13	*At1g03850*	CAGGATCACCAAAGCCAACA	AAGGGATAGAAATAAATAAGAAGGCAAC	La Camera *et al.*, 2011
AtPAL1	*At2g37040*	CAAGGTTTTCACGGCGATTT	CCGTTCCACTCGTTGAGACA	This work
AtPAL2	*At3g53260*	AAGGCTGTGCTTCCAAAGGA	CGTTCCAAGCTCTTCCCTCA	This work
AtiASK	*At5g26751*	CTTATCGGATTTCTCTATGTTTGGC	GAGCTCCTGTTTATTTAACTTGTACATACC	Gachon and Saindrenan, 2004
